# Analysis of *C9orf72* repeat expansions in Georgian patients with Amyotrophic lateral sclerosis (ALS)

**DOI:** 10.12688/f1000research.138436.1

**Published:** 2023-09-07

**Authors:** Mariam Kekenadze, Clarissa Rocca, Valentina Turchetti, Sara Nagy, Nana Kvirkvelia, Shorena Vashadze, Eka Kvaratskhelia, Maia Beridze, Rauan Kaiyrzhanov, Henry Houlden

**Affiliations:** 1 Tbilisi State Medical University, Tbilisi, 0141, Georgia; 2 Department of Neuromuscular Disorders, UCL Queen Square Institute of Neurology, University College London, London, England, UK; 3 Department of Neurology, University Hospital Basel, University of Basel, Basel, Basel-Stadt, Switzerland; 4 Tbilisi State University, Tbilisi, Georgia; 5 Batumi Shota Rustaveli State University, Batumi, Georgia

**Keywords:** ALS, MND, Gene, C9orf72, Georgia, DNA, Genetics, Genomics

## Abstract

**Background:** Amyotrophic lateral sclerosis (ALS) is a fatal progressive neurodegenerative disorder that affects the upper and lower motor neurons. Several genetic risk factors have been identified in the past decade with a hexanucleotide repeat expansion in the *C9orf72* gene being the most significant. However, the presence of *C9orf72* repeat expansion has not been examined in the Transcaucasian region, therefore we aimed to analyze its frequency in Georgian patients with ALS.

**Methods:** We included 64 self-reported Georgian patients with ALS from different parts of the country, fulfilling the Gold Coast criteria. To investigate the presence of an expanded GGGGCC hexanucleotide repeat in the non-coding region of the *C9orf72* gene, we performed Repeat-Primed PCR (RP-PCR).

**Results:** In total, 64 sporadic and two familial ALS cases were identified. Patients were aged 26 to 84 years with a mean age of 58.3 years at disease onset. Bulbar onset was observed in 21.88%, upper limb onset in 34.38%, and lower limb onset in 43.75% of the patients. Frontotemporal dementia (FTD) fulfilling the Strong criteria was diagnosed in seven patients (10.94%). *C9orf72* repeat expansion was detected in only one case using RP-PCR; the patient had a family history of dementia.

**Conclusions:** Our results indicate that *C9orf72* hexanucleotide expansion does not belong to the major genetic risk factor of ALS in Georgian patients. Further genetic studies in a bigger study population are needed to reveal the genetic causes of ALS in the Transcaucasian population.

**List of Abbreviations**
ALS: Amyotrophic Lateral Sclerosis
FTD: Frontotemporal dementia
LL-ALS: Lower Limb onset Amyotrophic Lateral Sclerosis
LMN: Lower Motor Neuron
PBP: Progressive Bulbar Palsy
PLS: Primary Lateral Sclerosis
PMA: Progressive Muscular Atrophy
RP-PCR: Repeat-Primed Polymerase Chain Reaction
TSMU: Tbilisi State Medical University
UCL: University College London
UL-ALS: Upper Limb onset Amyotrophic Lateral Sclerosis
UMN: Upper Motor Neuron

## Introduction

“Does it take place through simple propagation, extending gradually across the neuroglia?”. ^1^ This is what French neurologist J. M Charcot questioned regarding the disease development of amyotrophic lateral sclerosis (ALS) almost 150 years ago. Although the pathogenesis of ALS is still unknown, extensive studies have revealed important genetic risk factors in the past decade. *C9orf72* (#MIM 105550), *SOD1* (#MIM105400), *TARDBP* (#MIM612069 *,* #MIM612069 *)*, and *FUS* (#MIM608030) are the most frequently mutated genes that have been shown in ALS, ^2^^,^^3^ with the hexanucleotide repeat expansion in *C9orf72* being the most significant and accounting for 30–50% of familial and 7% of sporadic ALS cases in the European population. ^2^^,^^3^ However, the genetic basis of ALS has not been investigated in the Transcaucasian region. Therefore, we aimed to determine the frequency of *C9orf72* repeat expansion in Georgian patients with ALS.

## Methods

In total, 64 self-reported ethnically Georgian patients with ALS have been included in the study. There was no prior dataset of ALS patients in Georgia, nor epidemiological data about the disease, despite considering small size of Georgian population and rarity of the disease worldwide, we estimated that at least 50 participants would be scientifically significant number for this research. Ethical approval was obtained from Tbilisi State Medical University (TSMU) ethics committee (Date: 8 ^th^ June 2020, approval no. N3-2020/80) and University College London (UCL) institutional board (Short Title:IGC, CI:Prof H Houlden, Sponsor EDGE ID:146653,IRAS Approval Number:310045, Protocol V1.12 22.06.2019). Before the study, written informed consent was obtained from all subjects or their legal representatives in cases where participants were unable to write and sign the form.

Patients were recruited via phone during the years of 2019-2023 and the database of the First University Clinic of TSMU as well as of collaborative clinics were utilized for the study. Participants were contacted first of all to get information if they were still alive or not, and ask if they would agree on genetic testing in the future. No information was collected at that point, nor blood samples, before ethical approval was granted and consent taken from participants.

Diagnosis of ALS was based on the new Gold Coast criteria, incorporating progressive motor impairment documented by history or repeated clinical assessment, preceded by normal motor function, and the presence of upper and lower motor neuron dysfunction in at least one body region, or lower motor neuron dysfunction in at least two body regions, most importantly excluding other diseases. ^4^ Patients were reevaluated according to the Gold Coast criteria, however prior diagnosis and assessments were done by involved clinics. Patients diagnosed with conditions such as spinal muscular atrophy, Kennedy disease, monomelic amyotrophy, Hirayama syndrome, or multifocal motor neuropathy were excluded from the study, there were no other exclusion criteria. Also, clinical symptom studies were conducted using the Mayo Clinic Laboratory Neurological Questionnaire. Patients were distributed into clinical subtypes of ALS: typical, progressive muscular atrophy, primary lateral sclerosis, and progressive bulbar palsy. Furthermore, based on site of onset they were divided into three groups: bulbar onset, lower limb onset, upper limb onset. Cognitive changes were assessed using Addenbrooke’s Cognitive Examination Scale (ACE III) and the Frontal Behavioral Questionnaire, Frontotemporal dementia (FTD) was diagnosed according to the Strong critiera, ^5^ and the patient’s quality of life was assessed by ALSFRS-R.

Venous blood samples were collected in Georgia by MK, 5 ml venous blood samples were taken from the median vein of the forearm, EDTA k2 Vacutainers were used for storage and transferred to UCL Queen Square Institute of Neurology, Neurogenetics Laboratory for further research. The genomic DNA of the included subjects was extracted from whole blood using the Promega ReliaPrep™ Blood gDNA Miniprep System using manufacturers instructions.

**Figure 1.**
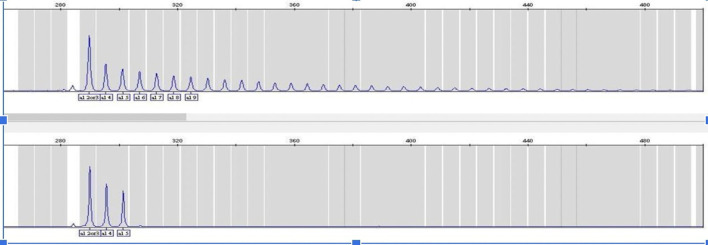
RP-PCR targeting the GGGGCC repeated hexanucleotide the plot in the top panel shows results from a positive control with the expanded repeats, and the bottom panel shows results from one of the non-expanded Georgian cases. This figure is an original figure produced by the authors for this article. RP-PCR, Repeat-Primed PCR.

To investigate the presence of an expanded GGGGCC hexanucleotide repeat in the non-coding region of the *C9orf72* gene, we performed Repeat-Primed PCR (RP-PCR) in all patients and in three positive controls. The primers and thermocycling conditions used for the assay have been previously described. ^6^ RP-PCR is able to determine whether an expanded allele is present in an individual, in which case a characteristic stutter pattern is seen ^7^ ( Figure 1).

All primers were used with the same molar concentrations. A PCR Mastermix was prepared by mixing 12.5 μl of Amplitaq Gold 360 Master Mix (ThermoFisher), 9.5 μl of 5M Betaine (ThermoFisher), 1 μl of 10 pmol/μl FAM labelled Forward primer (5′-TGTAAAACGACGGCCAGTCAAGGAGGGAAACAACCGCAGCC-3′), 1 μl of 10 pmol/μl Reverse primer (5′-CAGGAAACAGCTATGACC-3′), 1 μl of 10 pmol/μl repeat specific reverse primer (5′-CAGGAAACAGCTATGACCGGGCCCGCCCCGACCACGCCCCGGCCCCGGCCCCGG-3′) and 1 μl of 100 ng/μl DNA. Samples were amplified with an initial heat denaturation of 95°C for 10 minutes, followed by 10 cycles of 95°C for 30 seconds, 58°C for 2 minutes, 72°C for 2 minutes, and then 25 cycles of 95°C for 10 minutes, followed by 10 cycles of 95°C for 30 seconds, 58°C for 2 minutes, 72°C for 2 minutes with a 20 seconds increase per cycle. The final extension step was 72°C for 7 minutes. The PCR was run on a 9700 Block, at ramp speed 9600. After PCR, 1 μl of the reaction product was added to a mix with 9.2 μl of Formamide (Roche) and 0.1 μl of GeneScan 500 LIZ Size Standard (ThermoFisher). After a denaturation step at 95°C for 5 minutes, samples were analyzed using the ABI 3739 Genetic Analyser. Data were analyzed with the GeneMapper (RRID:SCR_014290) software (v. 4.0, Applied Biosystems).

## Results

Patients with ALS were aged 26 to 84 years with a mean age of 58.3 years at disease onset. In total, 63.8% of the patients were 50–69 years old. A total of 51% of the patients were male, 49% were female with a male-to-female ratio of 1:1.

**Figure 2.**
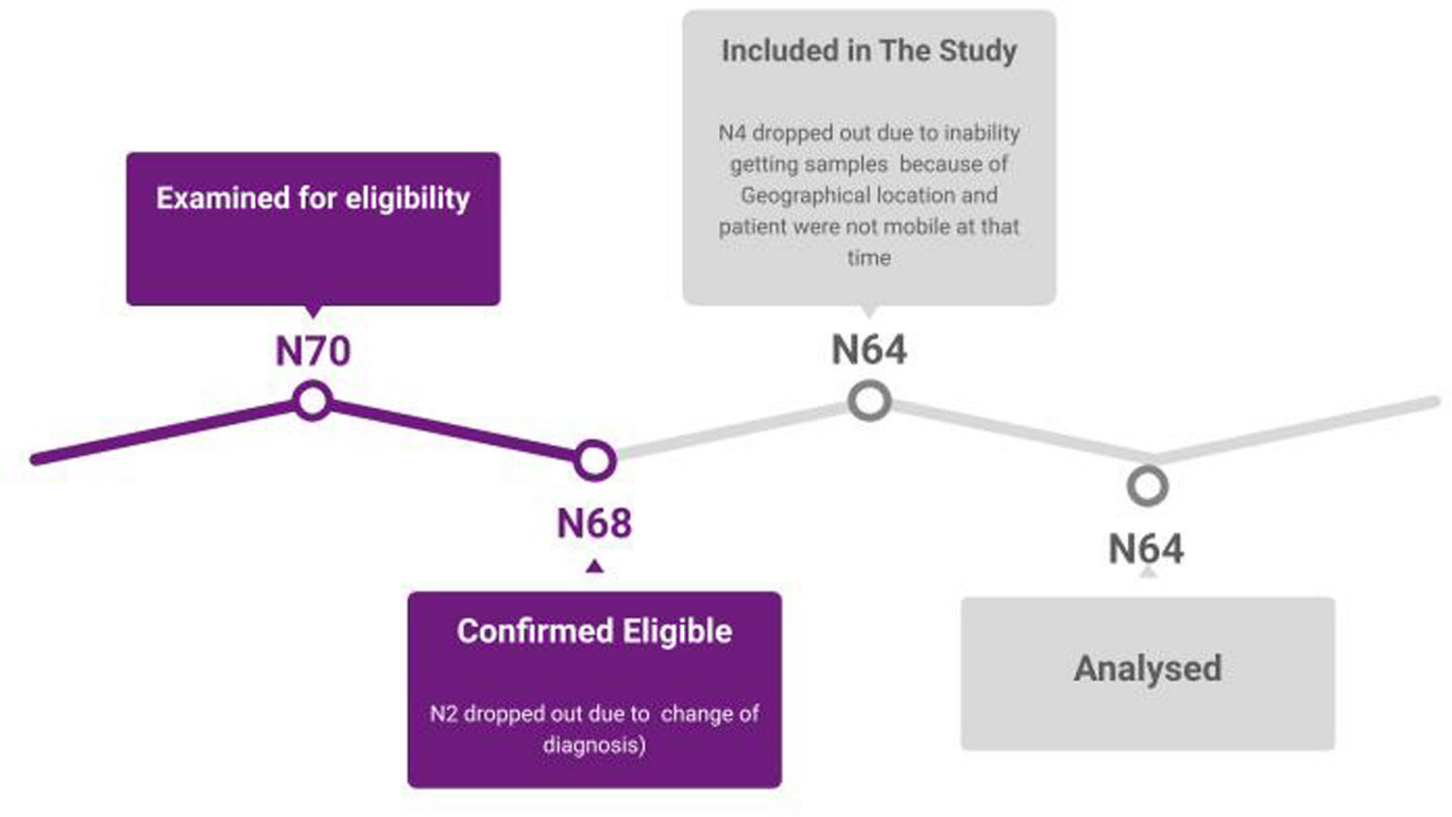
Representation of the number of participants at each stage. This figure is an original figure produced by the authors for this article.

After initial examination of 70 patients for eligibility, two patients were not confirmed to be eligible in the first stage, due to misdiagnosis of ALS, one of them had myasthenia gravis (MG) with anti-MUSK antibodies and the second patient had SMA (Spinal muscular atrophy) type 4. Four patients were not included in the study after being confirmed eligible, due to geographical conditions researchers were not able to get samples, and patients were not mobile, thus it was impossible for them to be transferred to the hospital ( Figure 2).

Bulbar onset ALS was observed in 21.3%, upper limb onset (UL-ALS) in 38.3%, and lower limb onset (LL-ALS) in- 40.4% of the patients ( Table 1). FTD fulfilling the Strong criteria was diagnosed in seven patients (10.94%). Two patients (3.13%) have been identified to have familial ALS (FALS) based on family history. ^8^

**Table 1.** Numbers and percentages of different ALS phenotypes in Georgian patients according to neuronal level, site of onset, and presence of ALS/FTD.

Phenotypic variant	UMN	LMN	n	%
Neuronal level				
Typical ALS	+	+	60	93.7
PLS	++	-	1	1.56
PMA	-	++	3	4.69
PBP	+	+	0	0
Site of onset				
Bulbar ALS			14	21.88
UL-ALS			22	34.38
LL-ALS			28	43.75
Mill’s (hemiplegic) variant			0	0
Flail Arm			0	0
Flail Leg			0	0
Presence of FTD				
ALS/FTD			7	10.94

ALS, amyotrophic lateral sclerosis; UMN, upper motor neuron; LMN, lower motor neuron; PLS, primary lateral sclerosis; PMA, progressive muscular atrophy; PBP, progressive bulbar palsy; UL-ALS, upper limb onset ALS; LL-ALS, lower limb onset ALS; FTD, frontotemporal dementia. + typical to a variable degree; ++ primary feature, - not a feature.

We screened all patients for GGGGCC hexanucleotide expansions between two 5′ non-coding exons of the *C9orf72* locus using RP-PCR. We used a reliable assay that confidently differentiates between positive and negative cases by detecting up to 40 repeats, thus categorizing them as pathogenic expansions. An accurate number of repeats in each allele can be detected in the negative cases. The repeat size in the general population has been observed to vary between two to 30 for healthy individuals, while affected people present at least one expanded allele with repeats ranging between 30 to several hundred hexanucleotides, ^6^^,^^7^ please see Figure N1 for comparison After performing RP-PCR, GGGGCC expansion was observed in only one patient. Most of our cases presented a homozygous two-repeats expansion. The mean expansion in our cohort was 2+3.11 repeats (Allele1 2-2, Allele2 2-12). ^9^

## Discussion

**Figure 3.**
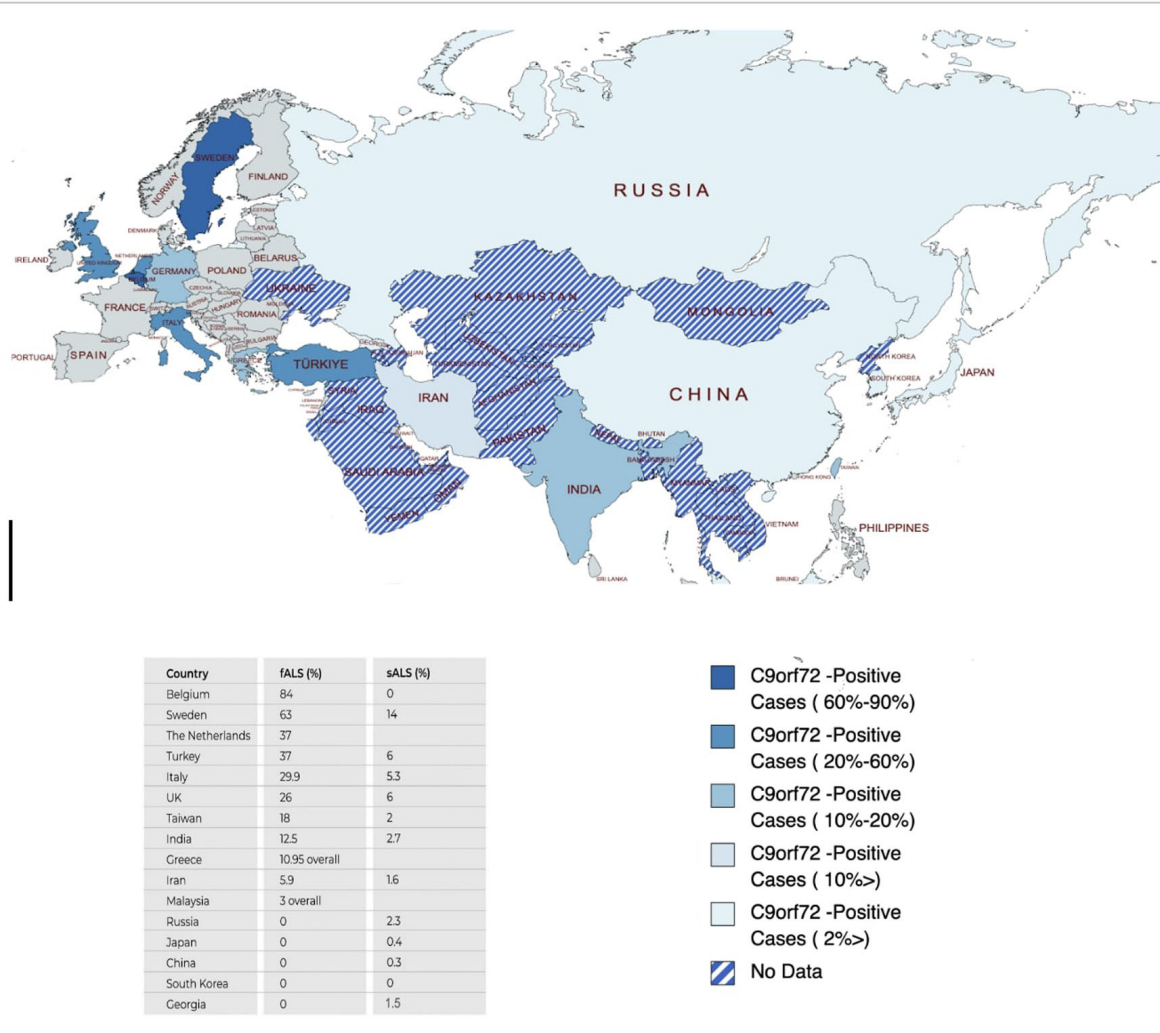
Eurasia map depicting c9orf-ALS plus cases across Eurasia colored according to prevalence. The bottom part shows the list of c9orf72 sALS and fALS cases in Eurasian countries reported before. ^10^^,^^12^^–^^25^ This figure is an original figure produced by the authors for this article. ALS, Amyotrophic lateral sclerosis; sALS, sporadic ALS; fALS, familial ALS.

A pathogenic repeat expansion in the non-coding region of the *C9orf72* gene has been described to be the most common risk to develop familial ALS. However, most studies have been performed in European study populations ^10^^–^^13^ and little is known from non-European countries. Based on these, a significant variation in c9orf-ALS frequency and distribution throughout Eurasia can be observed, with east Asian populations experiencing lesser cases, and India and Taiwan being exceptions ( Figure 3).

In our study, we aimed to investigate the frequency of c9orf-ALS in Georgian patients. However, from our 64 patients only one tested positive for pathogenic GGGGCC repeat expansion, and the patient demonstrated bulbar onset ALS, with a history of severe dementia. Our results possibly indicate a different genetic background and the presence of distinct risk factors for ALS in this ethnic group. The Georgian geography with its isolation and small population size, particularly in the highlands, might have led to the bottleneck effect and enhanced genetic differentiation seen in our data.

However, our results are limited due to the small cohort size. Further, the rare existence of the pathogenic repeat expansion could also be due to a previous single founder mutation. Smith *et al.* (2013) identified a haplotype that proves this point, that all massive GGGGCC hexanucleotide repeat expansion mutations—identified within intron 1 of *C9orf72*—carriers arose from a single common founder.13The most sensible explanation would therefore be that the expansion mutation arose on just one occasion in the European population. The results could further be explained by a close link between Georgian and Asian genetic pools, however, researchers reported that Caucasian groups were much closer to European than to West Asian groups with respect to mtDNA, opposite to be true for the Y chromosome, indicating a predominantly West Asian influence. ^26^

## Conclusions

Further genetic studies in a larger cohort are needed to confirm our results and to reveal genetic risks for ALS in the Transcaucasian population.

## Ethics approval and consent to participate

Ethical approval was obtained from Tbilisi State Medical University (TSMU) ethics committee and University College London (UCL) institutional board. All experiments were performed in accordance with WMA declaration of Helsinki – Ethical principles for medical research involving human subjects. Informed consent was obtained from all participants. All participants consented in written form to participate in the study. All authors attest that the participants were aware of the study’s purpose, risks, and benefits.

## Data Availability

Figshare: ALS Patients GEORGIA, https://doi.org/10.6084/m9.figshare.23731674. ^8^ This project contains the following underlying data:
- The spreadsheet data for all participants and outcomes underlying the Results section of the paper and Table 1. Figshare: RP-PCR Traces, https://doi.org/10.5522/04/23661813. ^9^ This project contains the following underlying data:
- The raw data of the genetic testing (RP-PCR Traces). Data are available under the terms of the Creative Commons Attribution 4.0 International license (CC-BY 4.0).
