# Supplementary material for: Analysis of C9orf72 repeat expansions in Georgian patients with Amyotrophic lateral sclerosis (ALS)
Source: F1000Res. 2024 Mar 6;12:1113. Originally published 2023 Sep 7. [Version 2] doi: 10.12688/f1000research.138436.2 (PMC10924727; doi:10.12688/f1000research.138436.2)
Supplement: Peer review history

# Reviewer report: Reviewer response for version 2

**DOI:** 10.5256/f1000research.163149.r253260

**Published:** 2024-03-09

**Authors:** Naoki Suzuki

**Affiliations:** 1 Tohoku University Graduate School of Medicine, Sendai, Japan

The authors appropriately improved their manuscripts.

Is the work clearly and accurately presented and does it cite the current literature?

Yes

If applicable, is the statistical analysis and its interpretation appropriate?

Yes

Are all the source data underlying the results available to ensure full reproducibility?

Yes

Is the study design appropriate and is the work technically sound?

Yes

Are the conclusions drawn adequately supported by the results?

Yes

Are sufficient details of methods and analysis provided to allow replication by others?

Yes

Reviewer Expertise:

Neurology

I confirm that I have read this submission and believe that I have an appropriate level of expertise to confirm that it is of an acceptable scientific standard.

# Reviewer report: Reviewer response for version 1

**DOI:** 10.5256/f1000research.151632.r238668

**Published:** 2024-01-19

**Authors:** Naoki Suzuki

**Affiliations:** 1 Tohoku University Graduate School of Medicine, Sendai, Japan

The report focuses on investigating the frequency of C9ORF72 mutations in ALS cases in Georgia, an area that has not been previously studied. This report holds considerable significance.

 Abstract:

 In the abstract, it is mentioned that there are 64 self-reported patients; however, in the Results section, it is specified as 64 sporadic and 2 familial cases. To avoid confusion, please correct the total count to 66.

 Methods:

 In the Methods section, it is stated as "ethnically Georgian." Is it acceptable to assume that there are no individuals with mixed ethnic backgrounds, such as half or quarter Georgian? Additionally, basic demographic information about Georgia's population would be appreciated.

 Results:

 For cases with C9ORF72 expansion, one instance with a family history of dementia is noted. It would be beneficial to provide details on the type of dementia and its progression.

 Participants:

 The statement indicating that scientifically significant interpretations can be made with 50 or more participants is made. Could you please provide the basis for this claim?

 Table 1:

 In Table 1, PBP (Progressive Bulbar Palsy) is listed as zero, while Bulbar onset ALS includes 14 cases. Please clarify the definition of PBP and how Typical ALS is defined.

 Figure 3:

 Concerns are raised about the data from the Nordic region in Figure 3, as the percentages appear unusually high. Please verify the accuracy of the literature sources.

 Typo：a single common founder. 37Themost sensible

Is the work clearly and accurately presented and does it cite the current literature?

Yes

If applicable, is the statistical analysis and its interpretation appropriate?

Yes

Are all the source data underlying the results available to ensure full reproducibility?

Yes

Is the study design appropriate and is the work technically sound?

Yes

Are the conclusions drawn adequately supported by the results?

Yes

Are sufficient details of methods and analysis provided to allow replication by others?

Yes

Reviewer Expertise:

Neurology

I confirm that I have read this submission and believe that I have an appropriate level of expertise to confirm that it is of an acceptable scientific standard, however I have significant reservations, as outlined above.

# Reviewer report: Reviewer response for version 1

**DOI:** 10.5256/f1000research.151632.r218006

**Published:** 2023-11-07

**Authors:** Karri Kaivola

**Affiliations:** 1 University of Helsinki, Helsinki, Finland

In their manuscript titled ”Analysis of C9orf72 repeat expansions in Georgian patients with Amyotrophic lateral sclerosis (ALS)”, Kekenadze *et al.* assess using repeat-primed PCR the *C9orf72* hexanucleotide repeat expansion frequency in 64 ALS patients from Georgia. The *C9orf72* hexanucleotide repeat expansion is the most common genetic risk factor in populations of European descent, but not in other populations.

 Even though the study population is small, it is enough to rule out C9orf72 expansion as a common cause of ALS in Georgia as only one patient carried the mutation. The knowledge gained from this study is pretty much covered in the previous sentence, but the study is methodologically acceptable. There are minor things to address:

1. The introduction is very short and sweet. I was hoping more background on the C9orf72 expansion prevalence in nearby regions and what is known about ALS genetics prior to this study in Georgia. These questions were partly covered at the start of the Methods and Figure 3, I would bring some of that information to Introduction.
2. Also would be useful to know, is the Georgian population homogenous and does the study cohort represent the whole population or just some part of it?
3. In Methods the authors write “we estimated that at least 50 participants would be scientifically significant number for this research.” Estimated how and what does scientifically significant number mean? Is this based on e.g. C9orf72 expansion frequencies in neighboring countries?
4. In Results after listing excluded cases, the authors could repeat the final sample number, even though it is in Figure 2.
5. 1st sentence of 2nd paragraph in Discussion: “c9orf-ALS” is a bit colloquial, I would use the full gene name c9orf72.
6. There is a stray citation number 13 in discussion in sentence “identified within intron 1 of C9orf72—carriers arose from a single common founder. ^13^”
7. The C9orf72 prevalence in Figure 3 seems unbelievably high in Belgium and Sweden (63-84% of fALS cases). I would urge the authors to reassess the evidence for these numbers, as all the numbers I have seen from robust reports have been considerably smaller.
8. In Figshare: ALS Patients GEORGIA table, the full dates of births of the patients are visible. Given the rarity of the disease, this makes the patients very identifiable, and I strongly urge the authors to remove such personally identifiable information and replace them with e.g. age. They could also add the C9orf72 allele lengths to the table.
9. The single founder hypothesis of C9orf72 expansion is very contested ^1^, I would refrain from making statements about a single founder.

In conclusion, this is a small study that fills a small gap in knowledge, but the study is generally well made and presented.

Is the work clearly and accurately presented and does it cite the current literature?

Yes

If applicable, is the statistical analysis and its interpretation appropriate?

Yes

Are all the source data underlying the results available to ensure full reproducibility?

Yes

Is the study design appropriate and is the work technically sound?

Yes

Are the conclusions drawn adequately supported by the results?

Yes

Are sufficient details of methods and analysis provided to allow replication by others?

Yes

Reviewer Expertise:

Neurogenetics

I confirm that I have read this submission and believe that I have an appropriate level of expertise to confirm that it is of an acceptable scientific standard.

## References

[1] : Large C9orf72 hexanucleotide repeat expansions are seen in multiple neurodegenerative syndromes and are more frequent than expected in the UK population. *Am J Hum Genet* .2013;92(3) : 10.1016/j.ajhg.2013.01.011 345-53 10.1016/j.ajhg.2013.01.011 23434116 PMC3591848

# Response

**Published:** 2023-11-15

**Authors:** Mariam Kekenadze

**Affiliations:** Neurology, Tbilisi State Medical University, Tbilisi, Georgia

Dear reviewer, Many thanks for your immense effort in reviewing our article, all the comments are much appreciated and looked at in detail. We would like to respond to some of them.

 1. Georgia is located in the Caucasus, neighboring countries are Armenia, Azerbaijan, Turkey, and Russia. After a thorough search of the literature using various languages, we were not able to find any relevant information about c9orf72 expansion prevalence in Armenia and Azerbaijan, however, there are some data available from Turkey and Russia, demonstrating low incidence in later countries, we depicted following in Eurasia map, Figure 3. Furthermore, there have not been any studies, including genetics, in the ALS population prior to this one in Georgia, indicating the significance of this research.

 2. There is sparse information regarding the genetic architecture of the Georgian population, therefore it is difficult to conclude the homogeneity of the population. The study indeed represents the whole Georgian population, samples were gathered from different parts of Georgia, and results are published in different article: https://pubmed.ncbi.nlm.nih.gov/37166887/

 3. We concluded that scientifically significant results would represent identifying a similar number of ALS patients in the Georgian population size according to worldwide incidence/prevalence of the disease. Unfortunately, there is no data available regarding c9orf72 gene expansion prevalence in ALS patients in neighboring countries.

 4. We agree with the reviewer.

 5. C9orf72 positive cases in familial ALS in Belgium and Sweden, we used the following article: Smith BN, Newhouse S, Shatunov A, et al.: The C9ORF72 expansion mutation is a common cause of ALS+/-FTD in Europe and has a single founder. Eur. J. Hum. Genet. 2013; 21(1): 102–108, who reported these findings. 

 6. Thank you for pointing out the issue with privacy in Figshare, we will fix it.
